# AI-Powered Chest X-Ray for Diagnosing Pulmonary Tuberculosis in County and Township Health Care Facilities in Yichang: Retrospective, Real-World Study

**DOI:** 10.2196/83041

**Published:** 2025-12-01

**Authors:** Wenjie Jiang, Hao Zhang, Zhili Li, Xinli Jiang, Jiamei Shao, Xuelin Yang, Jingjie Xiong, Ping Zhou, Hui Zhang, Hongsheng Wang, Jianxing Yu, Xiaoyou Su, Ye Wang, Jianhua Liu, Zhongjie Li

**Affiliations:** 1 School of Population Medicine and Public Health, Chinese Academy of Medical Sciences and Peking Union Medical College, 31 Bei Ji Ge San Tiao, Beijing, 100730, China, 86 010-65120552; 2 State Key Laboratory of Respiratory Health and Multimorbidity, Beijing, China; 3 Key Laboratory of Pathogen Infection Prevention and Control (Peking Union Medical College), Ministry of Education, Beijing, China; 4 Yichang Center for Diseases Control and Prevention, Yichang, China; 5 Beijing Chest Hospital, Capital Medical University/Beijing Tuberculosis and Thoracic Tumor Research Institute, Beijing, China; 6 The School of Public Health, Guilin Medical University, Guilin, Guangxi, China; 7 National Center for Tuberculosis Control and Prevention, Chinese Center for Disease Control and Prevention, Beijing, China; 8 Hospital for Skin Diseases, Institute of Dermatology, Chinese Academy of Medical Sciences and Peking Union Medical College, Nanjing, Jiangsu, China

**Keywords:** tuberculosis detection, chest X-ray, computer-aided detection, resource-limited settings, real-world study

## Abstract

**Background:**

In resource-limited areas, severe shortages of radiologists contribute to high rates of missed pulmonary tuberculosis (PTB) cases when relying solely on conventional chest X-ray (CXR). Although artificial intelligence–powered computer-aided detection (CAD) has proven effective in PTB diagnosis, its real-world performance remains underexplored.

**Objective:**

This study aimed to evaluate the real-world diagnostic yield of CAD technology as a triage tool for detecting PTB in primary health care facilities in high-burden areas.

**Methods:**

We conducted a retrospective paired-design diagnostic yield study using CXR images collected from 7 county- and 32 township-level health care facilities in Yichang city between 2022 and 2024 year. All images were retrospectively reprocessed with CAD software (JF CXR-1), and the original reports interpreted by radiologists at the time of patient admission were extracted. CAD and radiologist performances were compared using 2 primary evaluation indicators—diagnostic yield among diagnosed cases (DYD) and positive predictive value (PPV). Subgroup analysis (by region, age, sex, health care facility tier, and patient category) and sensitivity analysis were conducted to assess the robustness of the results.

**Results:**

Among 93,319 enrolled study patients, including 273 (0.3%) bacteriologically confirmed PTB cases, CAD demonstrated a substantially higher DYD (229/273, 83.9%) than radiologists (70/273, 25.6%), although the PPV was much lower (1.70% vs 10.31%). This high-sensitivity performance achieved an 85.5% (79,804/93,319) reduction (only 13,515 instead of 93,319 CXRs) in radiologist workload via selective review of CAD-positive images, without missing any radiologist-identified PTB cases. Furthermore, probability scores greater than 0.75 were a key threshold for identifying high-risk patients with PTB, and these patients were prioritized for radiologist review. Subgroup analysis further revealed that CAD outperformed radiologists in identifying PTB cases across all scenarios, despite some heterogeneity. CAD performance was significantly better in township-level medical facilities (DYD: 86.7%; PPV: 2%) than in county-level hospitals (DYD: 62.5%; PPV: 0.6%).

**Conclusions:**

CAD technology is valuable for detecting PTB in primary health care facilities. Combined with a tiered *artificial intelligence prescreening with selective human review* strategy, this approach effectively alleviates the workload of radiologists in resource-constrained regions, offering a scalable solution for tuberculosis prevention and control.

## Introduction

In 2023, an estimated 10.8 million new cases of tuberculosis (TB) were reported globally, among which 2.6 million went undiagnosed or unreported, contributing to ongoing transmission and high mortality rates [1-3]. Early screening and diagnosis are crucial for interrupting *Mycobacterium tuberculosis* transmission, but the current passive case-finding strategy, which relies on symptom-dependent access to health care, remains inefficient [4,5].

Chest X-ray (CXR) remains a common screening tool in primary health care facilities due to its ease of implementation, rapidity, and high acceptance [6]. However, the accuracy of CXR screening is influenced by radiologists' ability to identify pulmonary tuberculosis (PTB) cases [7]. The limited availability of well-trained radiologists in primary health care results in high rates of missed diagnoses for PTB, leading to delays in diagnosis and increased risks of disease transmission [8].

Artificial intelligence (AI)–assisted computer-aided detection (CAD) tools for CXR represent a promising innovation to enhance TB screening efficiency, diagnostic accuracy, and cost-effectiveness [9,10]. Currently, the performance of CAD tools has been shown to match or even surpass that of human experts, demonstrating high sensitivity and reducing diagnostic delays [11,12]. However, most of the existing research was conducted in controlled environments, such as those using standardized equipment and preselected patient cohorts [13,14]. The effectiveness of these tools in complex real-world settings has not yet been validated. Furthermore, CAD’s potential to automate initial screening offers a solution to address the shortage of health care personnel [15]. However, challenges remain, including controlling the false-positive rate (eg, misclassifying non-TB abnormalities) and uncertainties regarding the adaptability of these tools within tiered health care systems.

This study aimed to evaluate the diagnostic yield of CAD technology as a triage tool for detecting PTB in primary health care facilities in high-burden areas.

## Methods

### Study Design

Our study was conducted in 3 counties of Yichang city, Hubei province (Yidu, Zigui, and Changyang counties), a region characterized by a high PTB burden, with an overall incidence of PTB of nearly 100 per 100,000 population between 2022 and 2024. This study involved patients aged above 15 years (including outpatients and inpatients) who underwent CXR examinations at health care facilities due to symptomatic presentation. We used a paired diagnostic yield study design where each chest radiograph underwent 2 independent assessments—from an AI-powered CAD system and from clinical radiologists whose interpretations were the original reports from the time of patient presentation, thereby reflecting real-world conditions. To address the issue of multiple CXRs potentially occurring for the same patient during the study period, we established a stratified selection protocol—for non-PTB cases, the initial CXR examination was used and, for PTB cases, the CXR temporally closest to the confirmation date was selected. To reduce temporal misclassification bias between CXR acquisition and TB confirmation, we set a 6-month window period as the validity criterion.

### Details of the Retrospective Data

All CXR images were extracted in Digital Imaging and Communications in Medicine format from the Picture Archiving and Communication System-Radiology Information System of county- and township-level health care facilities in 3 study counties. The data collection period spanned from January 1, 2022, to December 31, 2024. The system also recorded patient demographics (gender and age) and the original radiology reports, including examination time, report date, the original radiological findings, and the diagnostic conclusions issued by the radiologists at the time of patient care. In this study, a suggestive PTB case was based on the content of the “diagnostic conclusions” field, which contained the radiologist’s ultimate clinical judgment on the CXR. If specific terms related to active PTB (such as “tuberculosis” or “pulmonary tuberculosis”) appeared in this field, the case was classified as “abnormalities suggestive of PTB.”

Data on PTB cases were retrieved from the Tuberculosis Information Management System (TBIMS). TBIMS is a population-based surveillance system that mandates reporting of all PTB cases, and the dataset used in this study spanned from January 1, 2022, to June 30, 2025, and included patient diagnosis type and confirmation date [16]. PTB cases in the TBIMS included clinically diagnosed PTB cases and bacteriologically confirmed PTB cases, in accordance with the diagnostic criteria and case classification guidelines issued by the Chinese national health authorities [17]. Clinically diagnosed cases were identified based on chest imaging, supplemented by clinical manifestations, immunology tests, and pathological examination or bronchoscopy. Bacteriologically confirmed cases were diagnosed based on a comprehensive assessment involving chest imaging, bacteriological tests (sputum smear microscopy for acid-fast bacilli or mycobacterial culture), molecular biological tests (such as Xpert MTB/RIF or Xpert Ultra), and lung tissue pathological examination.

### AI System for CXR Interpretation

For AI-based CXR interpretation, we used the JF CXR-1 software (version 2; JF Healthcare). It is an advanced AI system built on deep learning technology, primarily used for TB detection in CXRs with external validation support [18-20]. In 2022, the software was approved by China’s National Medical Products Administration as a class III medical device. The system generates probability scores ranging from 0 to 1, where higher values indicate a greater likelihood of active PTB. According to the manufacturer’s manual, scores exceeding 0.35 mandate additional diagnostic evaluation for TB. Consequently, this study used a threshold of >0.35 as the criterion for radiographic abnormalities suggestive of active PTB.

The following steps were performed on the software:

1. Digital Imaging and Communications in Medicine images were input into the system for preprocessing, which includes conversion to UINT8 format, contrast enhancement via histogram equalization, cropping to 1024×1024 resolution, conversion from single-channel to 3-channel format for model compatibility, and pixel normalization by subtracting the mean value of 128.
2. Feature maps were extracted through the ResNet34+fpn backbone network. These feature maps then triggered parallel dual-branch processing: branch 1 generates logit maps via a fully connected layer and outputs lesion, localizing heatmaps through a heatmap rendering module. Branch 2 compresses features via a global pooling layer, computes logits through a fully connected layer, and derives disease probability values via the Sigmoid function.

### Primary Indicators

We evaluated diagnostic performance using 2 primary indicators—diagnostic yield among all those diagnosed (DYD) and positive predictive value (PPV). DYD is defined as the proportion of confirmed patients with TB in whom the diagnostic tool (CXR interpreted by CAD or radiologists) successfully identified the disease [21]. Unlike a conventional sensitivity indicator, the DYD is calculated within a pragmatic clinical context. Its denominator includes all confirmed patients who underwent CXR as part of routine care, thereby accounting for real-world variables such as the time interval between imaging and confirmatory diagnosis. As a result, the DYD reflects the actual effectiveness of a tool in detecting TB among known patients under typical clinical circumstances, rather than its maximum theoretical accuracy under idealized research conditions. PPV is defined as the proportion of patients confirmed to have TB among all results identified as positive by a diagnostic tool (CXR interpreted by CAD or radiologists). It is crucial to acknowledge that this calculated PPV, based on bacteriological confirmation alone, should be interpreted as a lower-bound estimate. The true PPV is likely higher, as some CAD-positive cases not confirmed by bacteriology might represent occult TB missed by conventional diagnostics.

### Statistical Analysis

#### Descriptive Statistics

We summarized baseline characteristics of patients and presented categorical variables as frequencies (n) and proportions (%), and compared them using the chi-square test or Fisher exact test. Nonnormally distributed continuous variables were described as median (IQR) and analyzed using the Wilcoxon rank-sum test.

#### Diagnostic Performance

The ability of CAD and radiologists to identify PTB cases was evaluated using bacteriologically confirmed patients with PTB from TBIMS. To explore collaborative workflows between CAD and radiologists, performance was assessed under five distinct strategies: (1) radiologist alone, (2) CAD alone, (3) parallel test strategy (positive if either radiologist or CAD method flagged an abnormality), (4) serial test strategy (positive if radiologist and CAD both flagged an abnormality), and (5) CAD triage strategy, that is, CAD screening followed by radiologist verification (positive CAD results were reviewed by radiologists). For CAD-positive patients, the primary CAD scores were divided into 4 classes according to quartiles. Tests for linear trend between score quartiles and the distribution of patients with PTB (suspected by radiologists or confirmed) were conducted using Cochran-Armitage tests. Subgroup analyses were conducted to evaluate the DYD and PPV of the CAD system across diverse demographic and clinical variables. To assess the factors independently associated with these performance metrics, we constructed two separate multivariable logistic regression models:

1. For DYD, a logistic regression among the 273 confirmed PTB cases where the outcome was test positive (1=yes, 0=no). The odds ratios (OR) would represent the factors affecting the probability of detection, given a patient has PTB.

2. For PPV, a logistic regression among the 13,515 CAD-positive cases where the outcome was confirmed PTB (1=yes, 0=no). The OR would represent the factors affecting the probability of being a true positive, given that a patient was flagged abnormal by the AI.

### Sensitivity Analysis

Sensitivity analysis was performed to assess the robustness of CAD and radiologist performance in identifying PTB cases using CXR under different scenarios: (1) time window validation—assessment of different PTB confirmation intervals (3 mo and 9 mo vs the primary 6 mo window), (2) image selection validation—impact of image selection strategy (using the last CXR examination vs the initial CXR examination for patients without TB), and (3) case definition expansion—inclusion of clinically diagnosed PTB cases, to re-evaluate the performance of CAD and radiologists.

### Ethical Considerations

The study was approved by the Institutional Review Board of the Chinese Academy of Medical Sciences and Peking Union Medical College (CAMS&PUMC-IEC-2025‐04). The study retrospectively analyzed deidentified CXR images and PTB data obtained from routine clinical practice. A waiver of informed consent was received from the institutional review board. To protect privacy and confidentiality, all patient data were anonymized at the source, and the analysis was conducted exclusively on aggregated or coded datasets.

## Results

### Overview

**Figure 1.**
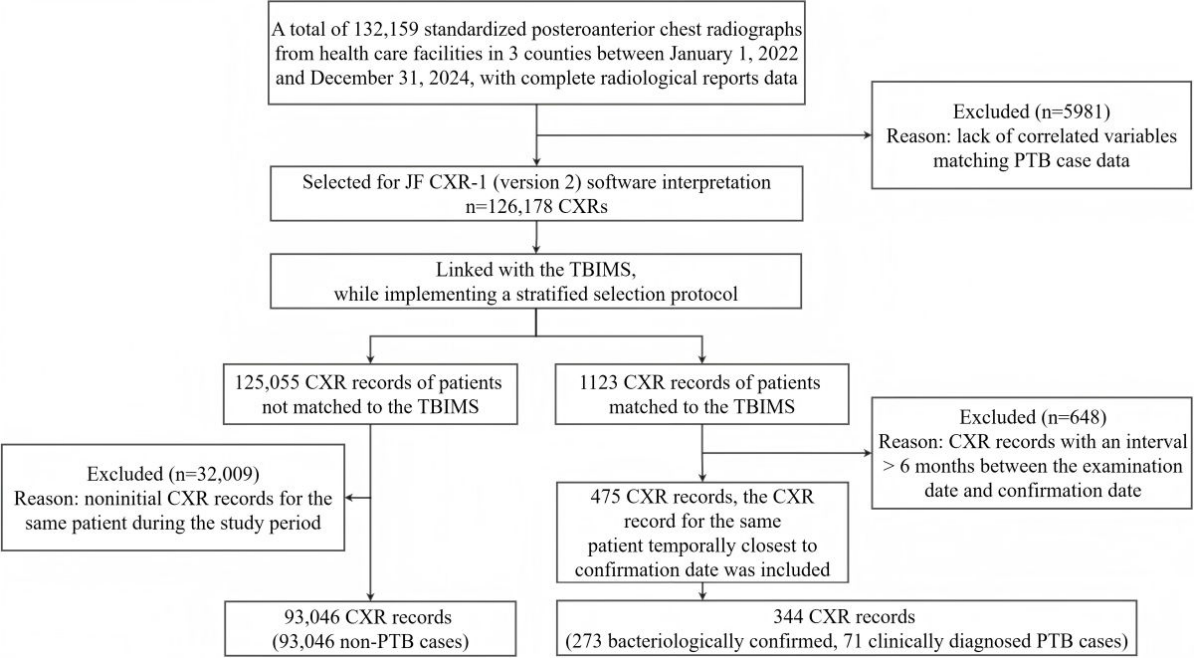
Flowchart depicting the study outline. For patients not matched to the TBIMS, we used their initial CXR records. For patients matched to the TBIMS, the CXR records conducted within 6 months preceding and temporally closest to their confirmation date were included. In total, 32.9% (369/1123) of CXR records were excluded because the time between CXR acquisition and confirmation exceeded 6 months, and 24.8% (279/1123) of CXR records were excluded as the CXR acquisition was performed later than the date of confirmation. CXR: chest X-ray; PTB: pulmonary tuberculosis; TBIMS: Tuberculosis Information Management System.

From January 2022 to December 2024, a total of 132,159 posteroanterior chest radiographs were collected from 7 county-level hospitals and 32 township health centers across 3 counties (Yidu, Zigui, and Changyang) in Yichang city, China. All images were accompanied by complete radiological reports. By linking PTB case data from the TBIMS, the final study population comprised 93,319 patients (non-PTB cases: n=93,046, 99.7%; bacteriologically confirmed PTB cases: n=273, 0.3%). The study outline is presented in Figure 1.

### Baseline Characteristics of Included Patients

A total of 93,319 patients (Figure 1) were included in this study. Characteristics of these patients are presented in Table 1. The median age of patients with PTB was higher than that of patients without PTB (69 years vs 60 years; *P<*.001), and male gender preponderance (70.7% vs 50.1%; *P<*.001) was observed. A higher proportion of patients with PTB came from township-level health care facilities compared to patients without PTB (88.3% vs 61.2%; *P<*.001). The proportion of patients with PTB from outpatient clinics was comparable with that of patients without PTB (29.7% vs 28.4%; *P*=.70).

**Table 1.** Baseline characteristics of included patients (N=93,319).

	Total patients (N=93,319)	Patients without PTB^a^ (n=93,046)	Confirmed patients with PTB (n=273)	*P* value
Region, n (%)				.001
Yidu county	35,020	34,941 (37.6)	79 (28.9)	
Zigui county	39,153	39,036 (42)	117 (42.9)	
Changyang county	19,146	19,069 (20.5)	77 (28.2)	
Age (y), median (IQR)	60 (50‐70)	60 (50‐70)	69 (60‐74)	<.001^b^
Age group (y), n (%)				<.001
15‐44	16,600	16,586 (17.8)	14 (5.1)	
45‐64	40,571	40,489 (43.5)	82 (30)	
65‐79	29,995	29,844 (32.1)	151 (55.3)	
≥80	6153	6127 (6.6)	26 (9.5)	
Sex, n (%)				<.001
Male	46,827	46,634 (50.1)	193 (70.7)	
Female	46,492	46,412 (49.9)	80 (29.3)	
Health care facility tier, n (%)				<.001
Township level	57,182	56,941 (61.2)	241 (88.3)	
County level	36,137	36,105 (38.8)	32 (11.7)	
Patient category, n (%)				.70
Outpatient	26,544	26,463 (28.4)	81 (29.7)	
Inpatient	66,775	66,583 (71.6)	192 (70.3)	

a PTB: pulmonary tuberculosis.

b *P* value was determined by Wilcoxon rank-sum test; others by chi-square test for categorical variables.

### Performance of PTB Detection: CAD Versus Radiologists

**Figure 2.**
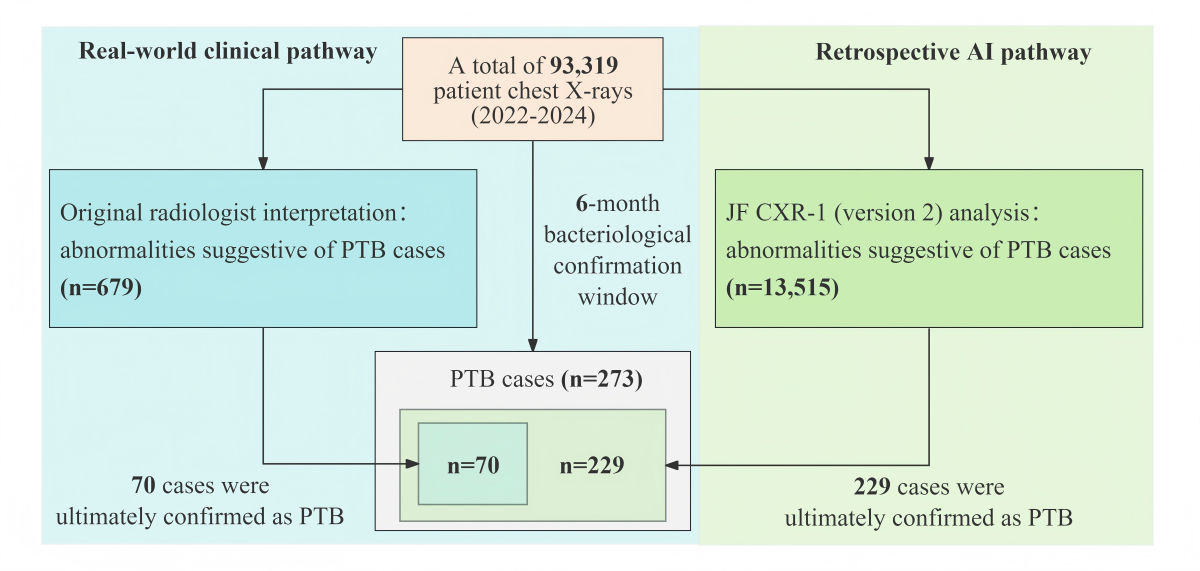
Comparative diagnostic yield for PTB: real-world clinical pathway versus retrospective AI analysis. The AI pathway identified a substantially higher number of cases (n=229), which included all 70 cases detected through the standard clinical pathway, including an additional 159 cases that were initially missed. AI: artificial intelligence; PTB: pulmonary tuberculosis.

Among 93,319 participants, 13,515 cases were identified as suspected PTB by CAD, with a positive rate of 14.5% (13,515/93,319), which significantly exceeded the positive rate of suspected PTB (679/93,319, 0.7%) identified by radiologists. Among the 273 patients with confirmed PTB, CAD achieved DYD of 83.9% (229/273), which was substantially higher than that of radiologists (70/273, 25.6%). CAD captured all 70 PTB cases detected by radiologists, demonstrating a low risk of missed detection. However, the PPV of CAD was only 1.7% (229/13,515), which was much lower than the radiologists’ PPV of 10.3% (70/679; Figure 2).

### Synergistic Performance of CAD-Assisted Radiologist Workflow

Strategy 3 required radiologists to interpret all 93,319 CXR images. It achieved a PPV of 1.7% (229/13,515), reflecting a marginal decrease compared to strategy 2 (PPV: 229/13,556, 1.7%). The DYD of strategy 3 was 83.9% (229/273), consistent with strategy 2. Strategy 4 similarly necessitated full interpretation of all CXRs. Its PPV increased to 11% (70/638), slightly exceeding strategy 1 (PPV: 70/679, 10.3%). The DYD of strategy 4 remained 25.6% (70/273), consistent with strategy 1. Strategy 5 enabled a radiologist workload reduction of 85.5% (1‐13,515/93,319) while maintaining a minimum system DYD of 25.6% (ie, finding all cases the radiologists originally found) and a potential DYD of 83.9% (if radiologists trusted the AI on all 229 positive cases; Table 2).

**Table 2.** Comparison of triage performance of different use strategies in pulmonary tuberculosis passive case-finding scenarios.

Strategy	Radiologist workload (chest X-ray reads), n/N (%)	DYD^a^, n/N (%)	PPV^b^, n/N (%)
Radiologist alone	93,319/93,319 (100)	70/273 (25.6)	70/679 (10.3)
CAD^c^ alone	0/93,319 (0)	229/273 (83.9)	229/13,515 (1.7)
Parallel test strategy^d^	93,319/93,319 (100)	229/273 (83.9)	229/13,556 (1.7)
Serial test strategy^e^	93,319/93,319 (100)	70/273 (25.6)	70/638 (11.0)
CAD triage strategy^f^	13,515/93,319 (14.5)	≥25.6% (up to 83.9%)^g^	N/A^h^

a DYD: diagnostic yield among diagnosed.

b PPV: positive predictive value.

c CAD: computer-aided detection.

d Positive if either the radiologist or CAD flagged an abnormality.

e Positive if radiologist and CAD both flagged abnormality.

f Positive if CAD flagged abnormality further confirmed by radiologist review.

g The final PPV or DYD depends on the radiologist’s confirmation of CAD-positive cases. Strategy 5 maintains a minimum system DYD of 25.6% (ie, finding all cases the radiologists originally found) and a potential DYD of 83.9% (if radiologists trusted the AI on all 229 positive cases).

h N/A: not applicable.

### Distribution of CAD Scores in Suspected PTB Cases

Among patients with abnormal CAD, each patient’s CAD score was classified into 4 subgroups by quartiles. The distribution of CAD scores was found to be linearly correlated with the proportion of suspected patients with PTB identified by radiologists and confirmed patients with PTB (*P* for trend <.001). Most confirmed patients (189/229, 82.5%) had CAD scores exceeding the 75% quantiles (Q75) threshold, which accounted for 34.2% (4618/13,515) of all CAD-positive patients (Table 3).

**Table 3.** Distribution of abnormal computer-aided detection (CAD) scores in suspected pulmonary tuberculosis (PTB) cases identified by radiologists and confirmed PTB cases.

CAD score (classified by quartiles)	CAD-positive patients (n=13,515), n (%)	Suspected PTB cases identified by radiologists^a^ (n=638), n (%)	Confirmed PTB cases^a^ (n=229), n (%)
0.35<score≤0.44	2828 (20.9)	13 (2)	6 (2.6)
0.44<score≤0.56	2995 (22.2)	26 (4.1)	11 (4.8)
0.56<score≤0.75	3074 (22.8)	70 (11)	23 (10)
score>0.75	4618 (34.2)	529 (82.9)	189 (82.5)

a Tests for linear trend between score quartiles and the distribution of patients with PTB (suspected by radiologists or confirmed) were conducted using Cochran-Armitage tests. The *P* value for suspected PTB cases identified by radiologists and confirmed PTB cases was <.001.

### Subgroup Analysis of PTB Detection Performance by CAD and Radiologists

**Figure 3.**
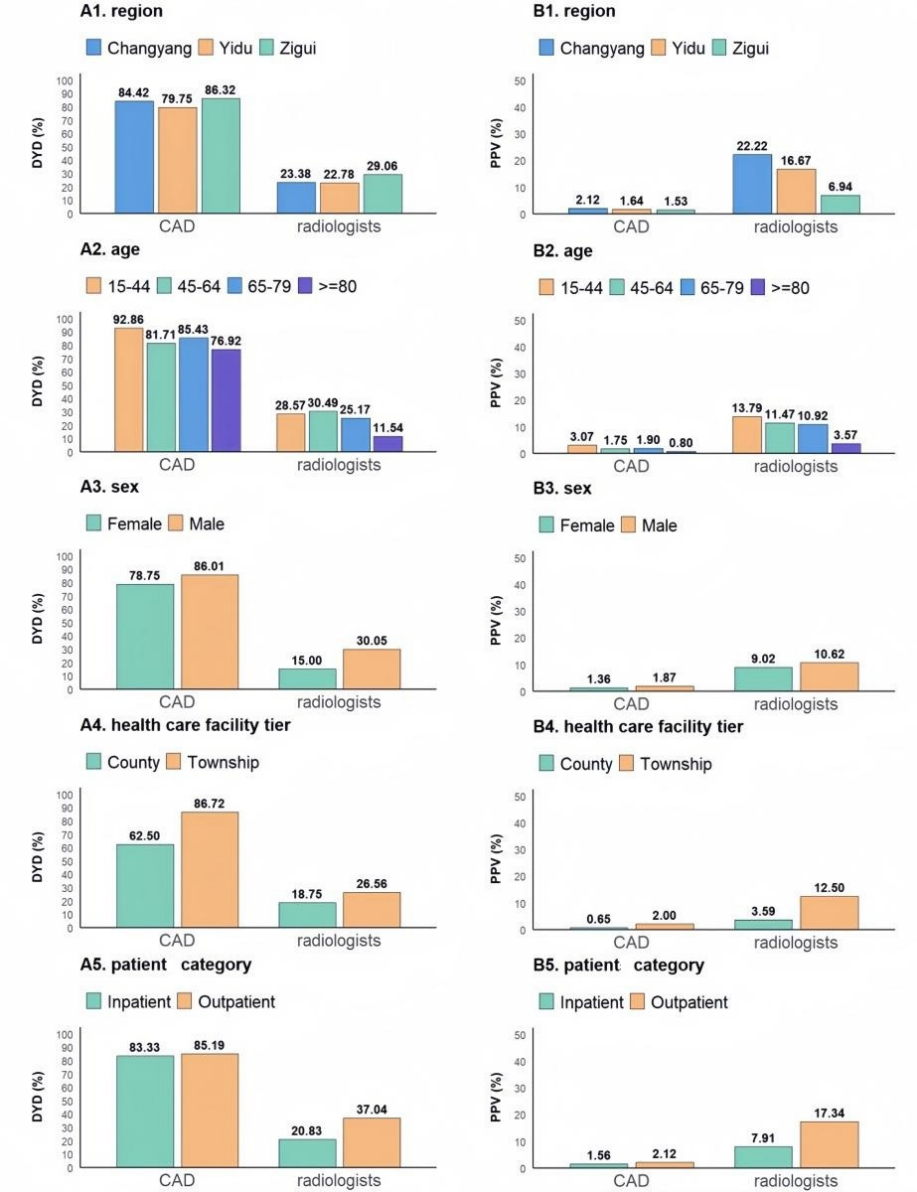
Comparison of DYD and PPV of CAD and radiologists by subgroups. Measures are disaggregated by A1 and B1: region, A2 and B2: age, A3 and B3: sex, A4 and B4: health care facility tier, and A5 and B5: patient category. CAD: computer-aided detection; DYD: diagnostic yield among diagnosed; PPV: positive predictive value.

CAD consistently demonstrated superior DYD compared to radiologists across all evaluated demographic and health care system classification variables (Figure 3). However, CAD showed significant variation in performance with respect to health care facility tier, performing poorer in the county-level group than in the township-level group (*P*<.001; Table S1 in Multimedia Appendix 1). Multivariate logistic regression also demonstrated that advanced age (≥80 y) groups were less likely to achieve high PPV (OR 0.21, 95% CI 0.10‐0.44; *P*<.001). No significant differences were observed between the sexes and patient categories (outpatient or inpatient).

### Sensitivity Analysis Results

During temporal window validation, shortening the PTB confirmation window to 3 months slightly increased CAD’s DYD to 86.6% (increased to 86.6% from 83.9%; Table S2 in Multimedia Appendix 1), whereas extending it to 9 months reduced DYD to 81.8% (decreased to 81.8% from 83.9%; Table S3 in Multimedia Appendix 1), indicating a minor influence of temporal factors on performance. Image selection validation (using final CXRs vs initial CXRs for non-PTB cases) revealed only subtle differences with unchanged core conclusions (Table S4 in Multimedia Appendix 1), confirming the reliability of the original strategy. When clinically diagnosed PTB cases (n=71) were included (Table S5 in Multimedia Appendix 1), the DYD of CAD decreased by only 2.8% (decreased to 81.1% from 83.9%), remaining significantly higher than that of radiologists (81.10% vs 25.58%). The DYD of radiologists decreased slightly (decreased to 25.6% from 25.6%). The consistent trends in both groups confirm that the relative advantage of CAD was not substantially affected.

## Discussion

### Principal Findings

This retrospective study evaluated the real-world performance of CAD in PTB detection using CXR interpretation. Among 93,319 patients analyzed from health care facilities, 273 (0.3%) were bacteriologically confirmed as PTB cases within 3 years. The results demonstrated that CAD outperformed radiologists in identifying PTB cases, detecting an additional 159 cases. Compared to *radiologist interpretation alone,* the strategy of *CAD screening followed by radiologist verification* not only enhanced PTB case identification but also reduced the workload for radiologists. A CAD score exceeding 0.75 was a key threshold for identifying high-risk patients with PTB, demonstrating high consistency with radiologists’ judgments and supported by statistical trends confirming significantly higher PTB risk with elevated scores. Notably, CAD exhibited superior diagnostic performance in township medical institutions compared to county-level hospitals, effectively overcoming inherent resource constraints.

This study observed that CAD demonstrated substantially higher DYD than radiologists in routine clinical practice. This comparison is inherently asymmetric, contrasting a specialized AI algorithm operating under optimal conditions against the daily performance of radiologists working in high-volume, multitasking environments [22]. Therefore, our findings are not intended to demonstrate that CAD is superior to radiologists in a direct competition. Instead, we aim to reveal and address a critical systemic flaw—the DYD of the current passive case-finding system in real-world clinical settings is alarmingly low (70/273, 25.6%). The significantly higher DYD (229/273, 83.9%) of CAD underscores its potential as a system-level triage tool, effectively bridging the sensitivity gap caused by resource constraints and radiologist fatigue.

The PPV of the CAD system is the most conservative estimate, as the CAD is almost certainly identifying true PTB cases that the system missed. However, excessive reliance on CAD-based diagnoses may potentially contribute to a higher volume of unnecessary follow-up procedures, leading to an inefficient allocation of medical resources. In resource-constrained primary care settings, CAD systems can serve as a triage tool for rapid PTB diagnosis [23]. By adopting a strategy of *computer-aided screening followed by selective radiologist review,* this approach optimizes the TB triage workflow. Such a stepwise process can effectively alleviate the workload of manual film reading while maintaining high sensitivity and reducing false-positive results [24]. Consistent with previous studies, CAD scores not only function as a diagnostic threshold but also possess predictive value for PTB risk based on continuous score variations [25]. CAD interpretation outcomes demonstrate high consistency with radiologists’ CXR readings, providing an objective reference for clinical decision-making [26-28].

We found that the diagnostic yield of CAD demonstrates significant variation across different levels of health care institutions, performing better in township-level facilities than in county-level hospitals. This performance disparity may be explained by several plausible factors related to the distinct patient populations and diagnostic pathways within the local health care ecosystem. We speculate that the reasons could be as follows: township-level facilities, serving as the primary contact point for a large volume of rural patients, may typically encounter cases at more advanced stages. Patients in these settings might tend to seek medical attention only when symptoms become severe and unmistakable, often presenting with more pronounced and characteristic radiographic abnormalities of PTB. These *classic* manifestations are likely to align well with the pattern recognition capabilities of current CAD systems. In contrast, county-level hospitals function as regional referral centers, potentially receiving a more complex case mix. This may include patients with early-stage or subtle disease, diagnostically challenging cases referred from township facilities, and a higher prevalence of nontuberculous pulmonary pathologies. Such complex and atypical presentations might approach the current detection limits of CAD algorithms. This finding underscores the importance of contextualizing CAD performance within the specific clinical environment and patient population it serves, and the proposed explanations warrant further investigation.

### Comparison With Prior Work

Previous research has centered on 2 PTB screening models—active screening in high-risk groups (eg, high-burden populations [19], cross-border migrants [29], and enclosed environments [30]) and passive screening in specialized facilities (eg, tertiary hospitals [11] and TB diagnostic centers [31]). This study pioneers PTB triage integration within China’s hierarchical health care system, offering novel evidence for AI deployment in primary care. While Kagujje et al [32] also studied primary settings, their focus was on *prior TB history* as an AI diagnostic confounder. The observed DYD of JF CXR-1 (229/273, 83.9%) aligns with high sensitivities reported previously (eg, Qin et al [20] reported a sensitivity of 86.4%). Compared with the sensitivity shown in clinical validation (eg, 78.9% in a study by Yang et al [12]), there is still a certain difference in performance in primary health care. Our proposed strategy of *artificial intelligence prescreening with selective human review* demonstrated its capability by reducing radiologist workload by 85.52%, aligning with the findings of Munjal et al [33]. On the basis of the TBIMS data, Xin et al [25] confirmed the effectiveness of CAD in individuals aged 65 years and above. This study further supplements evidence for the effectiveness of CAD in other application scenarios.

### Limitations

The 6-month interval between CXR acquisition and TB confirmation was defined primarily based on frontline physicians’ clinical experience, which indicates that the majority of diagnosable TB cases progress to a confirmable stage within this period. This window aligns with common diagnostic timelines in real-world practice and is methodologically consistent with previous studies (eg, Xin et al [25]). However, this exclusion may introduce selection bias, as the excluded cases with longer diagnostic delays may systematically represent patients with more subtle or early-stage disease that is inherently more challenging to detect. Consequently, the removal of these potentially *harder-to-diagnose* cases could lead to an overestimation of the DYD for both the CAD system and radiologists. Reassuringly, sensitivity analyses using both 3-month and 9-month windows consistently demonstrated the superior performance of the CAD system over radiologists, with only minor fluctuations in DYD values. This indicates that our primary conclusion remains robust despite the potential influence of this bias.

For CAD-positive cases not clinically diagnosed with PTB, the lack of subsequent verification makes it impossible to differentiate between true CAD false positives and occult TB cases missed by conventional diagnostics. The reported PPV of 1.7% is almost certainly an underestimate. The CAD system is likely identifying true active PTB cases that were missed by the current passive case-finding system, which relies on symptomatic presentation and subsequent bacteriological testing. Therefore, our findings not only demonstrate the high DYD of CAD but also point to a substantial number of potential PTB cases that merit further clinical investigation. This underscores the important role of CAD as a sensitive triage tool capable of uncovering the hidden burden of PTB in the community. Future studies with long-term clinical follow-up or additional diagnostic tests for CAD-positive individuals are needed to ascertain the true PPV.

This study was also limited to a unimodal radiographic input and did not integrate clinical symptom information. Since symptom profiles can influence both radiologists’ interpretations and the output of CAD systems, it would be valuable to evaluate whether diagnostic performance varies between symptomatic and asymptomatic subgroups. Although such a subgroup analysis fell outside the scope of this work, future investigations that incorporate multimodal data could help elucidate the relationship between symptomatic presentation and CAD performance, thereby contributing to the development of more clinically adaptive screening pathways.

### Conclusions

CAD dramatically improves the detection of PTB among symptomatic patients presenting to primary health care facilities, while substantially reducing radiologist workload through a selective review strategy focused on CAD-positive images with reference to the probability score. Meanwhile, CAD demonstrates significantly superior performance in township medical facilities compared to county medical facilities, providing validated evidence for implementing tiered diagnosis and treatment models in resource-limited regions.

## Supplementary material

10.2196/83041 Multimedia Appendix 1 Subgroup and sensitivity analyses.

## Abbreviations

AI: artificial intelligence
CAD: computer-aided detection
CXR: chest X-ray
DYD: diagnostic yield among all those diagnosed
OR: odds ratio
PPV: positive predictive value
PTB: pulmonary tuberculosis
TB: tuberculosis
TBIMS: Tuberculosis Information Management System
