# Supplementary material for: AI-Powered Chest X-Ray for Diagnosing Pulmonary Tuberculosis in County and Township Health Care Facilities in Yichang: Retrospective, Real-World Study
Source: J Med Internet Res. 2025 Dec 1;27:e83041. doi: 10.2196/83041 (PMC12670047; doi:10.2196/83041)
Supplement: Multimedia Appendix 1 [file jmir-v27-e83041-s001.docx]

**Multimedia Appendix 1.**

**Subgroup Analysis:**

**Table S1. Multivariable Logistic Regression Analysis of Factors Associated with Diagnostic Yield Among Diagnosed (DYD) and Positive Predictive Value (PPV).**

| Risk factors | DYD: PTB (n=273) | | | PPV: CAD-positive (n=13515) | | |
| --- | --- | --- | --- | --- | --- | --- |
|  | exp(B) | 95% CI | *P*-value | exp(B) | 95% CI | *P*-value |
| **Region** |  |  |  |  |  |  |
| Yidu County | 1 (ref) |  |  | 1 (ref) |  |  |
| Zigui County | 1.36 | 0.60~3.05 | .460 | 0.83 | 0.60~1.15 | .262 |
| Changyang County | 1.13 | 0.45~2.83 | .795 | 1.19 | 0.83~1.70 | .341 |
| **Healthcare Facility Level** |  |  |  |  |  |  |
| Township medical facility | 1 (ref) |  |  | 1 (ref) |  |  |
| County medical facility | 0.22 | 0.09~0.53 | **< .001** | 0.30 | 0.18~0.46 | **< .001** |
| **Visit type** |  |  |  |  |  |  |
| Outpatient | 1 (ref) |  |  | 1 (ref) |  |  |
| Inpatient | 0.98 | 0.43~2.10 | .953 | 0.88 | 0.66~1.20 | .407 |
| **Age (years)** |  |  |  |  |  |  |
| 15-44 | 1 (ref) |  |  | 1 (ref) |  |  |
| 45-64 | 0.23 | 0.01~1.46 | .193 | 0.49 | 0.27~0.94 | .022 |
| 65-79 | 0.29 | 0.01~1.77 | .265 | 0.51 | 0.29~0.97 | .027 |
| >=80 | 0.14 | 0.01~1.12 | .105 | 0.21 | 0.10~0.44 | **< .001** |
| **Sex** |  |  |  |  |  |  |
| Female | 1 (ref) |  |  | 1 (ref) |  |  |
| Male | 1.76 | 0.83~3.68 | .134 | 1.34 | 1.00~1.81 | .052 |

**Sensitivity Analysis:**

**Table S2. Comparison of triage performance of different usage strategies in PTB passive case finding scenarios. CXR: chest x-ray; CAD: computer-aided detection; DYD: diagnostic yield among diagnosed. PPV: positive predictive value. (3-month window: 231 PTB cases detected)**

| Strategy | | Radiologist Workload (CXR reads) | DYD (n/N) | PPV (n/N) |
| --- | --- | --- | --- | --- |
| 1 | Radiologist alone | 100% (93277/93277) | 28.57% (66/231) | 9.78% (66/675) |
| 2 | CAD alone | 0% (0/93277) | 86.58% (200/231) | 1.48% (200/13486) |
| 3 | Parallel test strategy ^a^ | 100% (93277/93277) | 86.58% (200/231) | 1.48% (200/13527) |
| 4 | Serial test strategy ^b^ | 100% (93277/93277) | 28.57% (66/231) | 10.41% (66/634) |
| 5 | CAD triage strategy ^c^ | 14.46% (13486/93277) | ≥28.57% (up to 86.58%) ^d^​ | N/A^d^ |
| ^a^ Positive if either radiologist or CAD flagged abnormality;  ^b^ Positive if radiologist and CAD both flagged abnormality;  ^c^ Positive if CAD flagged abnormality further confirmed by radiologist review;  ^d^ The final PPV/DYD depends on the radiologist's confirmation of CAD-positive cases, but the workflow ensures no loss of radiologist-identified cases while offering a major reduction in workload. | | | | |

**Table S3. Comparison of triage performance of different usage strategies in PTB passive case finding scenarios. CXR: chest x-ray; CAD: computer-aided detection; DYD: diagnostic yield among diagnosed. PPV: positive predictive value. (9-month window: 296 PTB cases detected)**

| Strategy | | Radiologist Workload (CXR reads) | DYD (n/N) | PPV (n/N) |
| --- | --- | --- | --- | --- |
| 1 | Radiologist alone | 100% (93342/93342) | 24.32% (72/296) | 10.57% (72/681) |
| 2 | CAD alone | 0% (0/93342) | 81.76% (242/296) | 1.79% (242/13528) |
| 3 | Parallel test strategy ^a^ | 100% (93342/93342) | 81.76% (242/296) | 1.78% (242/13569) |
| 4 | Serial test strategy ^b^ | 100% (93342/93342) | 24.32% (72/296) | 11.25% (72/640) |
| 5 | CAD triage strategy ^c^ | 14.49% (13528/93342) | ≥24.32% (up to 81.76%) ^d^​ | N/A^d^ |
| ^a^ Positive if either radiologist or CAD flagged abnormality;  ^b^ Positive if radiologist and CAD both flagged abnormality;  ^c^ Positive if CAD flagged abnormality further confirmed by radiologist review;  ^d^ The final PPV/DYD depends on the radiologist's confirmation of CAD-positive cases, but the workflow ensures no loss of radiologist-identified cases while offering a major reduction in workload. | | | | |

**Table S4. Comparison of triage performance of different usage strategies in PTB passive case finding scenarios. CXR: chest x-ray; CAD: computer-aided detection; DYD: diagnostic yield among diagnosed. PPV: positive predictive value. (For patients not included in TBIMS, the last CXR examination taken is extracted: 273 PTB cases detected)**

| Strategy | | Radiologist Workload (CXR reads) | DYD (n/N) | PPV (n/N) |
| --- | --- | --- | --- | --- |
| 1 | Radiologist alone | 100% (93319/93319) | 25.64% (70/273) | 10.92% (70/641) |
| 2 | CAD alone | 0% (0/93319) | 83.88% (229/273) | 1.65% (229/13850) |
| 3 | Parallel test strategy ^a^ | 100% (93319/93319) | 83.88% (229/273) | 1.65% (229/13885) |
| 4 | Serial test strategy ^b^ | 100% (93319/93319) | 25.64% (70/273) | 11.55% (70/606) |
| 5 | CAD triage strategy ^c^ | 14.84% (13850/93319) | ≥25.64% (up to 83.88%) ^d^​ | N/A^d^ |
| ^a^ Positive if either radiologist or CAD flagged abnormality;  ^b^ Positive if radiologist and CAD both flagged abnormality;  ^c^ Positive if CAD flagged abnormality further confirmed by radiologist review;  ^d^ The final PPV/DYD depends on the radiologist's confirmation of CAD-positive cases, but the workflow ensures no loss of radiologist-identified cases while offering a major reduction in workload. | | | | |

**Table S5. Comparison of triage performance of different usage strategies in PTB passive case finding scenarios. CXR: chest x-ray; CAD: computer-aided detection; DYD: diagnostic yield among diagnosed. PPV: positive predictive value. (Including Clinically Diagnosed PTB Cases: 344 PTB cases detected)**

| Strategy | | Radiologist Workload (CXR reads) | DYD (n/N) | PPV (n/N) |
| --- | --- | --- | --- | --- |
| 1 | Radiologist alone | 100% (93390/93390) | 25.58% (88/344) | 12.63% (88/697) |
| 2 | CAD alone | 0% (0/93390) | 81.10% (279/344) | 2.06% (279/13565) |
| 3 | Parallel test strategy ^a^ | 100% (93390/93390) | 81.10% (279/344) | 2.05% (279/13606) |
| 4 | Serial test strategy ^b^ | 100% (93390/93390) | 25.58% (88/344) | 13.28% (87/655) |
| 5 | CAD triage strategy ^c^ | 14.53% (13565/93390) | ≥**25.29**% (up to 81.10%) ^d^​ | N/A^d^ |
| ^a^ Positive if either radiologist or CAD flagged abnormality;  ^b^ Positive if radiologist and CAD both flagged abnormality;  ^c^ Positive if CAD flagged abnormality further confirmed by radiologist review;  ^d^ The final PPV/DYD depends on the radiologist's confirmation of CAD-positive cases, but the workflow ensures no loss of radiologist-identified cases while offering a major reduction in workload. A single case was identified by radiologists but not detected by the AI system. | | | | |
